# Physical Activity Increases the Total Number of Bone-Marrow-Derived Mesenchymal Stem Cells, Enhances Their Osteogenic Potential, and Inhibits Their Adipogenic Properties

**DOI:** 10.1155/2015/379093

**Published:** 2015-06-16

**Authors:** Monika Marędziak, Agnieszka Śmieszek, Klaudia Chrząstek, Katarzyna Basinska, Krzysztof Marycz

**Affiliations:** ^1^Faculty of Veterinary Medicine, University of Environmental and Life Sciences, Norwida 31 Street, 50-375 Wroclaw, Poland; ^2^Wroclaw Research Centre EIT+, Stablowicka 147 Street, 54-066 Wroclaw, Poland; ^3^Faculty of Biology, University of Environmental and Life Sciences, Kożuchowska 5b Street, 50-631 Wroclaw, Poland

## Abstract

Aging and sedentary lifestyle are common nowadays and are associated with the increasing number of chronic diseases. Thus, physical activity is recommended as one of three healthy behavior factors that play a crucial role in health prophylaxis. In the present study, we were interested whether physical activity influences the number and potential of bone-marrow-derived mesenchymal stem cells BMMSCs. In this study, four-week-old male C57Bl/6 mice were trained on a treadmill at progressive speeds over a 5-week period. Comparisons made between exercised (EX) and sedentary animal groups revealed (i) significantly higher number of MSCs in EX animals, (ii) elevated alkaline phosphatase (ALP) activity, (iii) increased level of osteopontin (OPN) and osteocalcin (OCL), and (iv) reduced marrow cavity fat. The results obtained support the thesis that EX may play a substantial role in the regeneration of mesenchymal tissues. Therefore, EX may represent a novel, nonpharmacological strategy of slowing down age-related decline of the musculoskeletal functions.

## 1. Introduction

Bone marrow (BM) contains various populations of developmentally early stem/progenitor cells, including endothelial progenitor cells (EPCs), very small embryonic/epiblast-like stem cells (VSELs), hematopoietic stem cells (HSCs), and mesenchymal stem cells (MSCs) [1]. MSCs are multipotent cells present in adult bone marrow, umbilical vein, and adipose tissue that possess capacity to proliferate and differentiate into various mesenchymal cell lineages, including adipocytes, chondrocytes, and osteocytes [2–4]. These stem cell populations are defined by the expression of various molecules, including CD90, CD105, and CD73, and the absence of markers such as CD34 or CD45 [5]. The combination of MSC differentiation potential and their paracrine effects makes them an attractive candidate for tissue repair and regenerative medicine. MSCs significantly influence the regenerative process through various mechanisms, for example, synthesis and secretion of membrane derived vesicles (MVs) [6, 7]. Increasing evidence has shown that the clinical application of bone-marrow-derived MSCs in some diseases leads to a successful regeneration of the damaged tissue [8]. The mechanisms increasing the number of MSCs in marrow are still poorly understood. Therefore, searching for efficient nonpharmacological methods that would significantly increase the total number of MSCs in the bone marrow may represent a helpful strategy for tissue regeneration.

Active lifestyle provides many physiological as well as psychological benefits. Many diseases, such as heart disease and type II diabetes, have been reported to be associated with limited physical activity. Thus, different types of exercises are recommended as a prophylactic measure, for example, highly aerobic endurance training program or moderate exercises. Each of them seems to act at different physiological levels, including improvement of immune and/or endocrine system. Although there has been little research conducted concerning the explanation of the mechanism of the positive effect of exercises on health, it has been demonstrated that exercises positively correlate with increased content of red blood cells [9, 10] and enhanced disease resistance* via* improved immune function [11]. In turn, moderate exercise has been reported as a factor that can boost immune function [12], while intense exercise can reduce immune response, causing a decrease in lymphocyte concentration, natural killer cell activity, and lymphocyte proliferation [13]. In addition, it was found that the exercise significantly reduces apoptosis and improves viability of osteocytes, as investigated using an osteopenic rat model [14]. It was observed in adolescent individuals that intensive exercises increased bone mass in the lumbar spine and femur [15]. Further, it appears that exercises, through biomechanical stimulation of the bone wall, may play a prevention role in bone resorption and formation [16].

The collective data suggests that physical exercises represent a nonpharmacological factor that positively affects longevity, prevention of metabolic diseases, and stem cell mobilization [17–19]. Therefore, in the present study, we have focused on (i) quantitative analysis of the total number of MSCs in the bone marrow, (ii) quantitative analysis of early and late markers of osteogenesis in osteoblasts precursors, and (iii) evaluation of femur mineralization process in mice exposed to endurance exercise training.

## 2. Materials and Methods

### 2.1. Animals and Exercise Training Protocol

Sedentary (SED) and exercise-trained (EX) C57Bl/6 mice (4 weeks old) were kept three per cage in an ultraclean facility on ventilated racks housed in the Animal Experimental Laboratory (Wroclaw Medical School, Norwida 34, Poland). Mice were maintained on a 12 h light-dark cycle at 22 ± 0.2°C. The experiment was conducted with the consent of the Local Ethics Committee for Animal Experiments. The animals were allocated to experimental groups (6 animals per group) divided into sedentary animals, which did not undergo physical activity (SE), and animals that exercised endurance (EX). Animals were exercise-trained (*n* = 6) 3 days per week (d/wk) (Monday, Wednesday, and Friday) for 5 weeks using an Exer 3/6 Treadmill (Columbus Instruments, Columbus, OH, USA). Afterwards, 5-wk progressive exercise protocol was used. The training started at 14 meters/min for 45 min (wk 1) and was gradually increased to 24 meters/min for 45 min (wk 5). The training portion of the protocol was always preceded by a 10 min warm-up at 10 meters/min, followed by a 5 min cool-down at 10 meters/min as described previously [20]. Sedentary control mice (*n* = 6) were exposed to the treadmill but not subjected to training. Sampling was carried out after the last training period. Bones (both femurs and tibiae) were collected, dissected of muscle and fat, and flushed with 1 mL of Iscove's modified Dulbecco medium (Sigma-Aldrich, Munich, Germany) with 2% FBS (Invitrogen, Carlsbad, CA, USA) for bone marrow cell isolation.

### 2.2. Preparation of MSCs for FACS

Murine MNCs (mononuclear cells) were isolated from the BM of pathogen-free, 4-week-old C57BL/6 mice. Bone marrow cell suspensions, isolated by flushing femurs and tibiae, were lysed in BD lysing buffer (BD Biosciences, San Jose, CA, USA) for 15 min at room temperature and washed twice in phosphate-buffered saline (PBS). The following anti-mouse antibodies (BD Pharmingen) were used for staining: anti-CD45 (allophycocyanin-Cy7, clone 30F11), anti-CD45R/B220 (PE, clone RA-6B2), anti-Gr-1 (PE, clone RB6-8 C5), anti-T-cell receptor-*αβ* (PE, clone H57-5970), anti-T-cell receptor-*γδ* (PE, clone GL3), anti-CD11b (PE, clone M1/70), anti-Ter119 (PE, clone TER-119), and anti-Ly-6A/E (also known as Sca-1, biotin, and clone E13-161.7, with streptavidin conjugated to PE-Cy5). MSCs (Sca-1+/Lin−/CD45−/CD31−/CD51+) were isolated by the multiparameter live-cell sorting (INFLUX, BD), as described previously [21]. Monoclonal antibodies (mAbs) used in this study were added at saturating concentrations. The cells were incubated for 30 minutes on ice with different anti-mouse monoclonal antibodies, washed twice, and resuspended in staining buffer at a concentration of 5 × 10^6^ cells per milliliter. Samples were analyzed on a LSR II flow cytometer (BD Biosciences, Mountain View, CA, US). The following anti-mouse mAbs were purchased from BD Pharmingen (San Diego, CA): Ly-GA/E (Sca-1, FITC, clone D7), lineage marker (CD45R (also known as B220), PE, clone RA3-6B2), Ly-6G/Ly-6C (PE, clone RB6-8C5), T-cell receptor b (PE, clone H57-597), T-cell receptor cd (PE, clone GL3), CD11b (PE, clone M1/70), Ter119 (PE, clone TER-119), CD51 (biotin, clone RMV-7, with streptavidin conjugated to PE-Cy5 as secondary Ab), CD31 (APC, clone 390), and CD45 (APC-Cy7, clone 30-F11). The results are representative of three independent experiments.

### 2.3. Multipotency Assay

Cells were seeded in 24-well plates at a concentration of 2 × 10^4^ cells per well and subsequently adipogenic, chondrogenic, and osteogenic differentiations were performed. Stimulation of MSCs was performed using StemPro Adipogenesis, Chondrogenesis, and Osteogenesis Differentiation Kits (Life Technologies, Poland) (StemPro Adipogenesis Differentiation Kit, StemPro Chondrogenesis Differentiation Kit, and StemPro Osteogenesis Differentiation Kit) according to the manufacturers' instruction. Differentiations began 24 h after inoculation and cells were cultured for 14 days. Media were changed every three days. The stage of adipogenic, chondrogenic, and osteogenic differentiation of MSCs was assessed using specific staining: Oil Red O (Sigma-Aldrich, Munich, Germany) for detecting neutral lipid deposits, Safranin O (Sigma-Aldrich, Munich, Germany) for glycosaminoglycans staining, and Alizarin Red (Sigma-Aldrich, Munich, Germany) for calcium deposits. Preparations were analyzed using Axio Observer A1 inverted microscope (Axio Observer A1, Carl Zeiss, Jena, Germany), while the documentation was made using Cannon PowerShot camera.

### 2.4. CFU-Fs Assay

Assays for colony-forming unit fibroblasts (CFU-Fs) were prepared according to the protocol described previously by Baker et al. [20]. MSCs derived from bone marrow (200 cells) of exercising (*n* = 6) and sedentary animals (*n* = 6) were plated in collagen-coated 35 mm tissue culture plates. The cultures were supplemented with *α*-MEM with 15% FBS, penicillin, and streptomycin, and the cells were maintained in culture for 14 d. Next, the cells were fixed with 4% paraformaldehyde for 10 min and subsequently stained with 0.5% crystal violet (Sigma-Aldrich, Munich, Germany) in 20% methanol for 5 min. Only colonies consisting of 20 aggregated cells or more were counted as CFU-Fs. All experiments were performed in triplicate.

### 2.5. Histology and Immunohistochemistry

Both humeri of all animals were fixed (4% formaldehyde in PBS, 48 h; Sigma-Aldrich), decalcified by EDTA (Osteosoft, Merck Millipore) at 37°C for 5 days, and rinsed with PBS. All samples were dehydrated in increasing ethanol gradient of 50%, 60%, 70%, 80%, 96%, and 100%. Then, the samples were embedded in paraffin, as previously described [22]. The samples were sectioned transversely (HM 340E, Microm) at a thickness of 10 *μ*m, dried, rehydrated in alcohol series, and stained with hematoxylin and eosin (Thermo Scientific, Lutterworth, UK). Next the samples were dehydrated and mounted under a coverslip. The slides were examined by light microscopy (Axio, Zeiss). The thickness of the endosteum was measured and normalized at photo area. For immunohistochemistry, 6 *μ*m paraffin-embedded sections were placed on adhesive plates and dried at 37°C for 24 hours. Samples were then deparaffinized in xylene and rehydrated in alcohol series with decreasing concentrations. The EnVision System (Dako) was used to visualize the antigen-antibody reaction. Rabbit anti-CD105 (Abcam, Cambridge, UK) primary monoclonal antibodies were used. All antibodies were diluted according to the manufacturer's instructions. Images were taken using Cannon PowerShot Camera.

Quantification of CD105 positive staining was performed using computer-assisted image analyses with a Java-based open source image processing software ImageJ v1.48c. Image analysis was performed on six randomly chosen previously captured images. The analysis included color threshold method, binary conversion, and measurement of the active areas using measure plugin and expressed as percentage of positive staining per image.

### 2.6. Osteogenic and Adipogenic Differentiation of MSCs

For osteogenesis, half of the MSC plates were used. After the first passage, the cells were placed in 24-well culture plates at a concentration of 1.5 × 10^5^ cells per well and cultured in osteogenic medium (StemPro Osteogenesis Differentiation Kit, Life Technologies). Osteogenic differentiation began 24 h after inoculation and was completed 21 days later. Culture media were changed every three days. In addition to osteogenic differentiation, adipogenesis assay was also performed. The cells were maintained in *α*-MEM, with 15% FBS, penicillin, and streptomycin, for 14 days. To quantify adipogenesis, cells were stained with Oil Red O (Sigma-Aldrich, Munich, Germany), eluted with isopropanol. Representative images were captured using Axio Observer A1 (Zeiss, Oberkochen). For quantitative analysis, absorbance was measured at 490 nm, following destaining with 100% ethanol for 20 min.

### 2.7. Scanning Electron Microscopy/Energy Dispersive X-Ray Spectroscopy (SEM-EDX) Analysis

Humeri were fixed in 2.5% glutaraldehyde for one hour, washed in distilled water, dehydrated in a graded ethanol series, sputtered with gold (ScanCoat 6, Oxford), and imaged using a scanning electron microscope (EVO LS15, Zeiss) at 10 kV of filament's tension. Additionally, specimens were prepared for EDX analysis according to the method described previously by our laboratory [23, 24]. Observations were performed at the filament's tension of 10 kV. The detection of mineralized matrix was performed using SEM combined with EDX (Bruker, Coventry, UK), by analyzing the surface distribution of calcium and phosphorus. The values obtained were presented as weight percentage (wt%).

### 2.8. Alizarin Red S Staining, Quantitative Alkaline Phosphatase (ALP), Osteocalcin (OCL), and Osteopontin (OPN) Assay

Alizarin Red S staining was performed to evaluate the formation of calcium deposits. Briefly, after a 21-day period of osteogenic differentiation, cells were fixed in 10% formalin for 1 h at room temperature, followed by washing with distilled water and incubation for 5 min in the working solution of Alizarin Red S. After staining, cells were washed three times in distilled water and calcium deposits were observed under an inverted microscope (Axio Observer A1, Zeiss). Photographs were acquired using Cannon PowerShot digital camera. For quantitative analysis of Alizarin Red staining, plates were incubated with 10% acetic acid for 30 min and then the contents of the wells were collected into 1.5 mL sample tubes, mixed, heated to 85°C for 10 minutes, cooled on ice for 5 minutes, and pH-neutralized with 10% ammonium hydroxide. After the spin-down (1200 ×g, 15 min), the absorption of the supernatant at 405 nm wavelength was measured using a microplate reader (SPECTROstar Nano, BMG Labtech). Extracellular activity of ALP was determined in the supernatants collected on the last day of cell incubation. The assay was performed using Alkaline Phosphatase Colorimetric Assay Kit (Abcam, Cambridge, UK), according to the protocol provided by the manufacturer. Experimental samples were prepared in duplicate and diluted twofold. A control sample of the background was included and the background was corrected by subtracting the values derived from the zero standards from all standards, samples, and the control sample.* p*-nitrophenyl phosphate (pNPP) was used as a substrate for phosphatase. The substrate was hydrolyzed into* p*-nitrophenol by alkaline phosphatase.

The reaction product was measured at 405 nm wavelength using a microplate reader (BMG Labtech). Sample readings were applied to the standard curve to obtain the amount of pNP generated by the ALP sample. Enzymatic activity was determined using the following formula: ALP activity (U/mL) = *A*/*V*/*T*, where (i) *A* is the amount of pNP generated by the samples (in nmol), (ii) *V* is the volume of sample added to the assay well (in mL), and (iii) *T* is the reaction time.

The levels of osteocalcin (OCN) and osteopontin (OPN) were determined in the supernatants after 21 d of cell culture. The medium was collected in three separate replicates. Prior to the protein level measurements, samples were thoroughly mixed. In order to determine the concentration of extracellular proteins, specific ELISA kits were used: (i) Rat Gla-Osteocalcin High Sensitive ELISA Kit (Takara Bio Europe, Saint-Germain-en-Laye, France) and (ii) Mouse/Rat Osteopontin Quantikine ELISA Kit (R&D Systems, UK). Quantitative determination of OCN and OPN was performed according to the manufacturers' instructions. The amount of proteins detected was expressed as a ratio of protein mass and supernatant volume (w/v).

### 2.9. Statistical Analysis

Statistical analysis was performed using Statistica 9.0 software (StatSoft Polska Sp. z o.o., Kraków, Poland). The significance of differences between the results obtained was evaluated using the unpaired *t*-test or one-way ANOVA with Fisher's post hoc test. The results were considered significant at *p* ≤ 0.05. All data were presented as mean ± SD.

## 3. Results

### 3.1. The Total Number of MSCs Isolated from Bone Marrow and Clonogenic Potential

**Figure 1:**
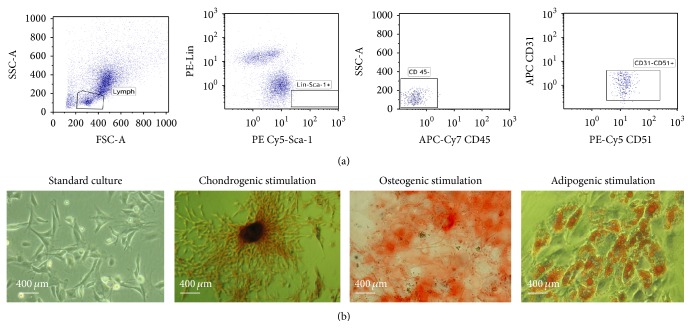
Analysis of MSC (a) phenotypes using flow cytometry. The FACS profile of separated Sca-1 positive and Lin-negative cells demonstrating CD31^negative^, CD45^negative^, and CD51^positive^ MSCs (a). (b) Morphology of MSCs cultured in DMEM medium supplemented with 10% of FBS (standard culture) and the results of its chondrogenic, osteogenic, and adipogenic stimulation. For visualization of glycosaminoglycans and calcium deposits specific Safranin O and Alizarin Red staining were used, respectively, while lipid droplets were stained using Oil Red O.

In order to confirm cells' mesenchymal origin the criteria for definition of MSCs were fulfilled: (i) typical plastic adherent growth; (ii) expression of MSCs markers and absence of surface hematopoietic markers; and (iii)* in vitro* differentiation potential toward chondrogenic osteogenic and adipogenic lineage (Figure 1).

**Figure 2:**
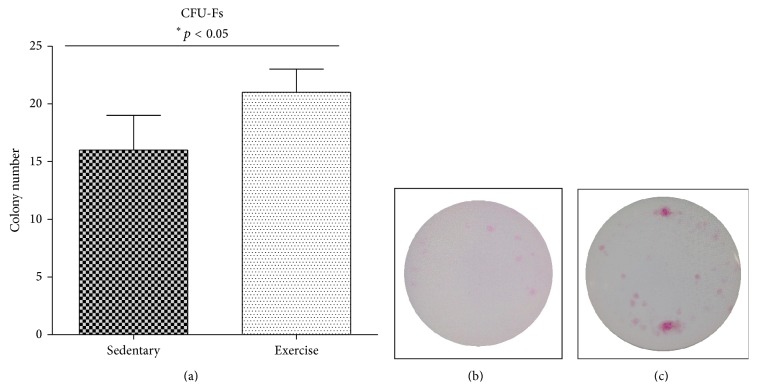
The number of MSC colony-forming units (CFU-Fs) (a). Representative images of stained cells collected from sedentary (b) and exercised (c) groups.

The number of colonies formed by MSC progenitors after 21 days of culture was significantly higher in endurance-trained animals in comparison to the sedentary group. Exercises resulted in the formation of 21 MSC colonies (21 ± 2; *p* ≤ 0.05) per well, while in sedentary group 16 colonies were observed (16 ± 3; *p* ≤ 0.05) (Figure 2).

**Figure 3:**
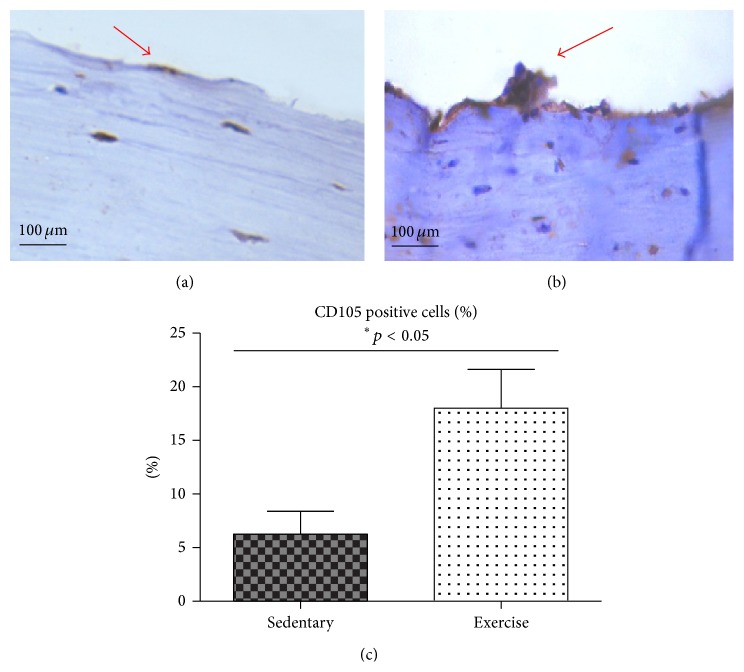
(a, b) Immunohistochemical stainning the CD105^+^ cells stained immunohistchemically in the sedentary group (a) and exercised (b) group. Positive reactions are indicated by red arrows. Quantification of CD105 positive cells; results representative of six randomly choosen pictures are expressed as mean ± SD (^*∗*^ *p* value < 0.05).

The number of MSCs isolated after endurance exercises was significantly elevated (4 ± 2*∗*10^6^; *p* ≤ 0.05), in comparison to the MSCs isolated from sedentary animals (1.8 ± 2*∗*10^6^; *p* ≤ 0.05). Moreover, the immunohistochemical staining to detect CD105^+^ cells revealed more intensive reaction in endurance-trained animals (Figures 3(b) and 3(c)) in comparison to the sedentary group (Figures 3(a) and 3(c)).

### 3.2. The Concentration of Calcium and Phosphorus in the Bone Wall

**Figure 4:**
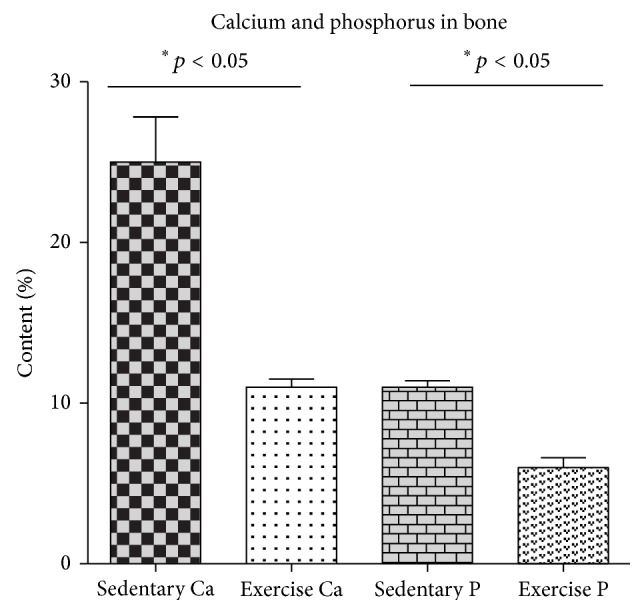
The concentration of calcium and phosphorus in the bone after endurance exercise training. Statistical significance (*p* < 0.05).

SEM-EDX analysis was performed to estimate the effect of EX on the bone mineralization. Quantitative EDX analysis demonstrated that the concentration of Ca and P in the bone wall of sedentary animals was 42% higher (26 wt% ± 6; *p* ≤ 0.05) when compared to the exercised group (11 wt% ± 2; *p* ≤ 0.05). The data are presented in Figure 4.

### 3.3. MSC Osteogenic Potential

**Figure 5:**
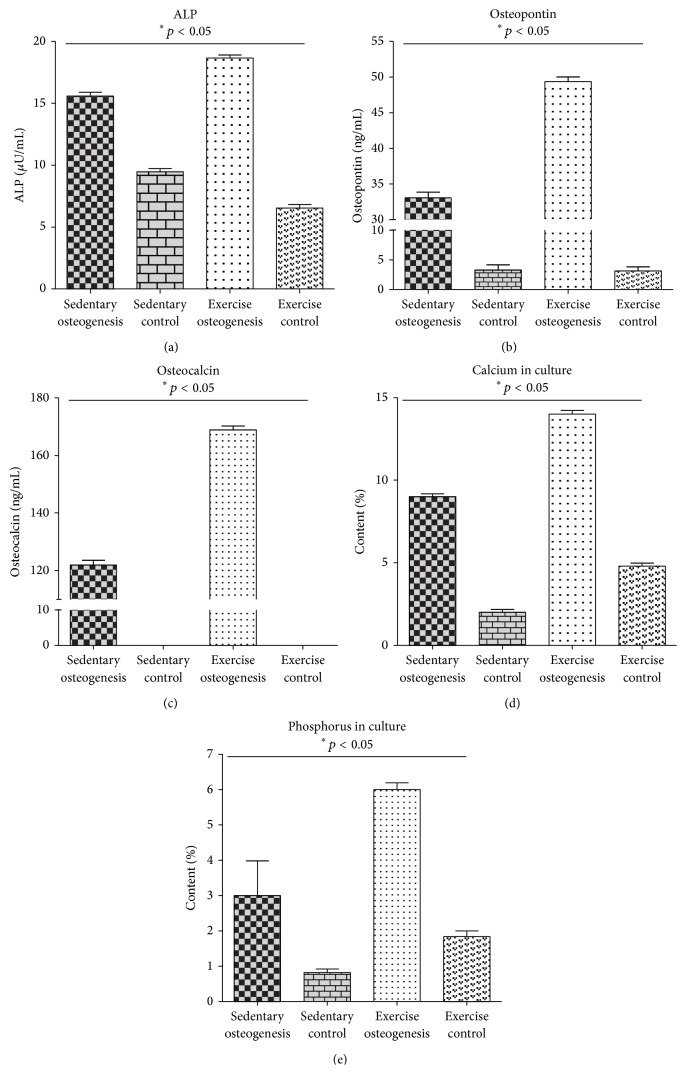
The activity of ALP (a) and the concentration of OPN (b), OCL (c), calcium (d), and phosphorus (e) measured on the 21st day of culture in osteogenic medium. Statistical significance (*p* < 0.05).

**Figure 6:**
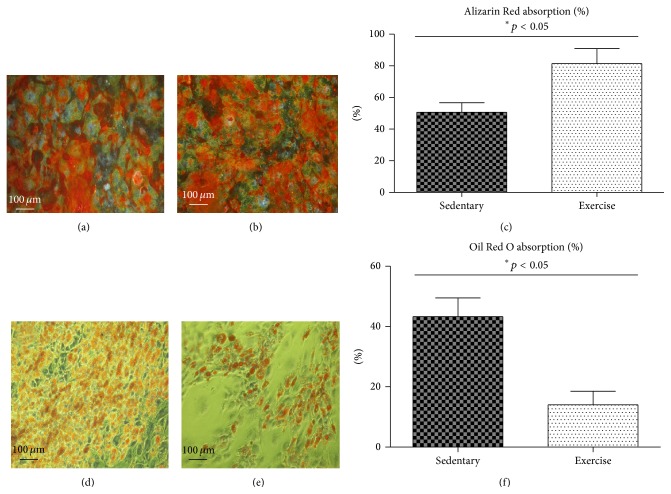
Alizarin Red staining of osteoblasts derived from MSCs collected from sedentary (a) and exercised (b) individuals and quantity of absorbed dye (c).(d, e, and f) Representative images of 21 d, Oil Red O stained and quantified, and differentiated MSCs isolated from sedentary (d) and exercise-trained (e) animals. Percentage of Oil Red O absorption (f) cells derived from sedentary and exercised animals. Statistical significance (*p* < 0.05).

A comparative analysis between both groups was performed to investigate whether EX exerted an effect on the osteogenic differentiation potential. We analyzed early and late markers of osteogenesis as well as Ca and P concentrations on the surface of osteoblast precursors. A significantly higher level of alkaline phosphatase (ALP) was detected in endurance-trained animals (19 uU/mL ± 1; *p* ≤ 0.05) when compared to the sedentary group (16 uU/mL ± 1; *p* ≤ 0.05) (Figure 5(a)). A similar correlation was observed in the level of osteopontin (OPN). The highest OPN level, equal to 48 ± 2 ng/mL, was recorded in endurance-trained animals, while in sedentary group it amounted to 32 ± 2 ng/mL (Figure 5(b)). This tendency was also retained with respect to osteocalcin (OCL) level in both groups investigated. Higher OCL level (170 ± 1.3 ng/mL; *p* ≤ 0.05) was detected in the exercised group, compared with the sedentary group (130 ± 1.4 ng/mL; *p* ≤ 0.05) (Figure 5(c)). Data obtained strongly correlated with quantitative analysis of Ca and P performed using SEM-EDX. A higher concentration of calcium (14 ± 1 wt%; *p* ≤ 0.05) and phosphorus (6 ± 2 wt%; *p* ≤ 0.05) was observed in the exercised group in comparison to the sedentary group, where Ca and P were equal to 9 ± 2 wt% and 3 ± 0.6 wt%, respectively (*p* ≤ 0.05) (Figures 5(d) and 5(e)). Alizarin Red staining revealed significant differences (*p* ≤ 0.05) in the MSC mineralization process in both groups tested. A considerably stronger reaction and the highest percentage of absorbed Alizarin Red were observed in exercised animals (81% ± 9) (Figures 6(b) and 6(c)) when compared to sedentary individuals (56% ± 3) (Figures 6(a) and 6(c)).

### 3.4. Reduced Number of Adipocytes in the Bone Marrow Cavity and Inhibition of MSC Adipogenic Potential

**Figure 7:**
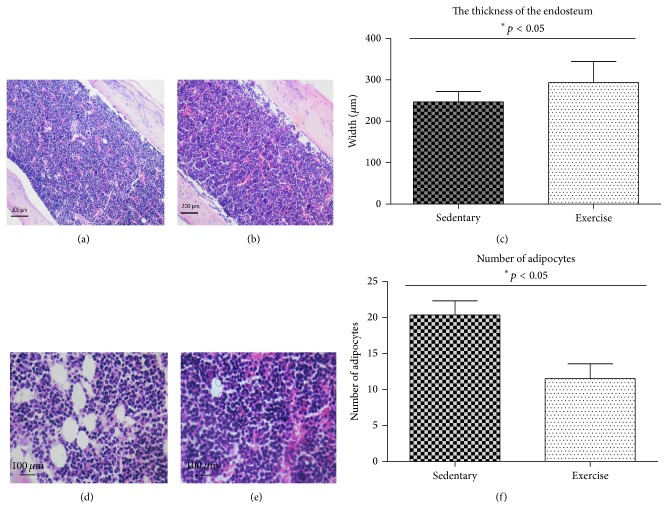
The morphology of the bone marrow cavity and endosteum in sedentary (a) and exercised animals (b). Representative images, taken in the central area of the marrow cavity, of bones stained with hematoxylin and eosin from sedentary (d) or exercise-trained animals (e). The thickness of murine endosteum in exercise-trained animals and sedentary group (c) and the total number of adipocytes in the bone marrow cavity in sedentary and endurance exercising animals (f). Number of adipocytes normalized on photo areas. Statistical significance (*p* < 0.05).

Picture of the marrow cavity was investigated using hematoxylin and eosin (H+E) staining. Larger deposits of fat droplets were observed in sedentary animals in the marrow cavity (Figures 7(a) and 7(d)) when compared to the exercising animals (Figures 7(b) and 7(e)). Quantitative evaluation of the total number of adipocytes in the marrow cavity revealed that sedentary animals had 55% more of adipocytes (20 ± 3; *p* ≤ 0.05) compared to the exercised animals (11 ± 2; *p* ≤ 0.05) (Figure 7(f)). Moreover, the thicker bone wall (328 *µ*m ± 36) was observed in exercising animals when compared to the control individuals (223 *µ*m ± 14; *p* ≤ 0.05).

The adipogenic potential of MSCs isolated from sedentary and endurance-trained animals was evaluated using standard MSC adipogenesis-inducing protocol. Oil Red O staining revealed more extensive adipogenesis in sedentary animals in comparison to the exercised group (Figures 6(d) and 6(e)). Additionally, it was found that the adipogenic differentiation potential of MSCs derived from endurance-trained animals was decreased based on the lower percentage of absorbed Oil Red O dye (17% ± 3; *p* ≤ 0.05) compared to sedentary group (46% ± 3; *p* ≤ 0.05) (Figure 6(f)).

## 4. Discussion

In recent years, much attention has been paid to the development of strategies aimed at slowing down aging process and its consequences. Aging and sedentary lifestyle contribute to the reduction of bone quantity and quality, decreased muscle mass and strength, and weakened postural stability, culminating in an elevated risk of skeletal fractures. Regenerative ability of an individual strongly correlates with the number of stem cells that can serve as a backup population for the repair/rejuvenation of damaged tissue [21, 24]. Therefore, seeking nonpharmacological methods that will increase the number of stem cells of different origin seems to be a major medical challenge.

Mechanical signals affect bone marrow-derived stem cell populations, particularly mesenchymal stem cells (MSCs), which has a significant impact on the bone and fat morphology [25]. In this study, we have shown that the endurance exercise training (EX) increases the total number of mesenchymal stem/progenitor cells (MSCs) in the bone marrow cavity. Moreover, in our previous study, we have demonstrated that EX increased the total number of VSELs circulating in PB and residing in BM. Thus, we could speculate that EX may have beneficial effects on the expansion of both early developmentally stem cells (VSELs) and stem/progenitor cells that might play an important role in tissue and organ rejuvenation. As previously reported, circulating stem cells are involved in repairing minor exercise-related tissue and organ injuries [25]. Moreover, we have found that EX enhances the osteogenic potential of MSCs which is consistent with the results of Baker et al. [20]. The latter author group reported that EX enhances hematopoiesis due to the increased endocrine signaling from skeletal muscleand remodeling of the medullary hematopoietic niche.

Given the fact that the bone niches are inhabited by MSCs, their number and differentiation potential appear to be a crucial factor in the context of body regenerative needs. MSCs present in bone marrow provide microenvironmental support for hematopoietic stem cells (HSCs) and can additionally differentiate into various mesodermal lineages [21, 26]. The present study demonstrates that the physical activity can increase the total number of bone-marrow-derived MSCs. An increased number of MSCs in the bone marrow was strongly correlated with a higher number of fibroblast colony-forming units (CFU-Fs) in exercised animals. Similar results were obtained by Baker at al. [20], who reported a higher number of CFU-Fs of MSCs in trained animals. In the current work we have found that EX decreases the amount of fat in the bone marrow cavity. We have noticed a lower number of adipocytes in the bone marrow cavity in endurance-trained individuals when compared to sedentary animals. Our data is consistent with findings of other research groups that have also observed a decreased number of adipocytes in exercised animals and humans [20]. It is well known that the fatty bone marrow has a negative effect on hematopoiesis. This is due to not only the occupation of marrow space, but also the protein factors released by adipocytes, such as neuropilin-1 [27], lipocalin 2 [28], or adiponectin [29], which can significantly impair hematopoietic proliferation.

In the perspective of stem cells' application in the clinical practice, it is important not only to increase hematopoiesis, but also to achieve and maintain an adequate amount and quality of bone tissue. Interestingly, in our study, osteoblast precursors (OBs) exhibited a higher osteogenic potential in endurance-trained animals when compared to the sedentary group. We have found a higher activity of alkaline phosphatase (ALP), an early marker of osteogenesis as well as elevated levels of late markers of osteogenesis, that is, osteopontin (OPN) and osteocalcin (OCL). Furthermore, it was found that endurance training enhanced the process of mineralization in OBs. Higher calcium (Ca) and phosphorus (P) levels were detected in OBs derived from MSCs of exercised animals, which highlighted the efficiency of osteogenesis* in vitro. *In contrast, a lower level of Ca and P was observed in the humerus of trained animals. This might be the result of Ca and P release to the peripheral blood during exercises, which has been previously reported by other authors [30]. Changes observed in differentiation potential as well as the mineralization process of OBs derived from EX animals might be the result of mechanical strain stimulus. It was reported that the mechanical forces applied to the bone during treadmill training program activated extracellular signal-regulated kinase pathway (ERK1/2), which resulted in a more mature osteogenic phenotype of MSCs [20]. Additionally, bone exposure for physical strain was shown to downregulate peroxisome proliferator-activated receptor (PPAR-*γ*), also known as the glitazone receptor (PPARG) that stimulates lipid uptake and inhibits adipogenesis [30, 31]. In our study, we observed a significantly lower number of lipid droplets in adipocyte precursors derived from MSCs in EX animals. This indicates the inhibitory effect of EX on the adipogenic potential of MSCs. Mechanical and physical stress, including exercises, might influence changes in cells actin organization. Actin cytoskeleton dynamics have been associated with the regulation of adipogenic and osteogenic differentiation. Signals forwarded from physical activity are transduced by integrins to the actin cytoskeleton to switch mechanical signals into biochemical pathways—from adipogenic to osteogenic differentiation [32–34].

The study performed by Menuki at al. [35] reported that climbing exercise enhanced MSC differentiation potential and bone remodeling. Another study demonstrated that the cyclic strain enhanced matrix mineralization in human mesenchymal stem cells (hMSCs).

## 5. Conclusions

In conclusion, we have shown that endurance training increases the total number of mesenchymal stem/progenitor cells in the bone marrow. Our results suggest that EX not only supports the process of hematopoiesis, but also significantly enhances the osteogenic differentiation potential of MSCs, with simultaneous inhibition of their adipogenic properties. The results support the thesis that endurance training can play a substantial role in the regeneration of mesenchymal tissues and thus represents a novel nonpharmacological manner of delaying age-related weakening of the musculoskeletal system.
